# Decreased plasma level of C-type lectin-like receptor 2 (CLEC-2) in patients with breast cancer

**DOI:** 10.7150/ijms.117190

**Published:** 2025-10-20

**Authors:** Chia-Chi Chen, Chin-Feng Hsuan, Teng-Hung Yu, Chia-Chang Hsu, Cheng-Ching Wu, Wei-Hua Tang, Wei-Chin Hung, Yung-Chuan Lu, Fu-Mei Chung, Yau-Jiunn Lee, Ching-Ting Wei

**Affiliations:** 1 Department of Pathology, E-Da Hospital, I-Shou University, Kaohsiung 82445, Taiwan.; 2 School of Medicine, College of Medicine, I-Shou University, Kaohsiung 82445, Taiwan.; 3 Department of Physical Therapy, I-Shou University, Kaohsiung 82445, Taiwan.; 4 Department of Occupational therapy, I-Shou University, Kaohsiung 82445, Taiwan.; 5 Division of Cardiology, Department of Internal Medicine, E-Da Hospital, I-Shou University, Kaohsiung 82445, Taiwan.; 6 Division of Cardiology, Department of Internal Medicine, E-Da Dachang Hospital, I-Shou University, Kaohsiung 807066, Taiwan.; 7 Division of Gastroenterology and Hepatology, Department of Internal Medicine, E-Da Hospital, I-Shou University, Kaohsiung 82445, Taiwan.; 8 Health Examination Center, E-Da Dachang Hospital, I-Shou University, Kaohsiung 807066, Taiwan.; 9 The School of Chinese Medicine for Post Baccalaureate, College of Medicine, I-Shou University, Kaohsiung 82445, Taiwan.; 10 Division of Cardiology, Department of Internal Medicine, E-Da Cancer Hospital, I-Shou University, Kaohsiung 82445 Taiwan.; 11 Division of Cardiology, Department of Internal Medicine, Ministry of Health and Welfare Yuli Hospital, Hualien 98142, Taiwan.; 12 Faculty of Medicine, School of Medicine, National Yang Ming Chiao Tung University, Taipei 112304, Taiwan.; 13 Division of Endocrinology and Metabolism, Department of Internal Medicine, E-Da Hospital, I-Shou University, Kaohsiung 82445, Taiwan.; 14 Lee's Endocrinologic Clinic, Pingtung 90000, Taiwan.; 15 Division of General Surgery, Department of Surgery, E-Da Hospital, I-Shou University, Kaohsiung 82445 Taiwan.

**Keywords:** Breast cancer, C-type lectin-like receptor 2, concentrations, overall survival

## Abstract

**Background:** C-type lectin-like receptor 2 (CLEC-2) is involved in platelet activation, tumor metastasis, and vessel differentiation, but its role in breast cancer remains unclear. This study examined the association between clinical status and plasma levels of CLEC-2 in patients with breast cancer.

**Methods:** Plasma CLEC-2 concentrations were measured using ELISA in breast cancer patients and control subjects. A total of 98 breast cancer patients and 98 age-matched control subjects were enrolled. All study participants were female.

**Results:** CLEC-2 concentrations were significantly lower in the breast cancer patients (231.2 [213.5-250.9] ng/mL) than in the controls (249.2 [235.8-263.4] ng/mL, p < 0.0001). Plasma CLEC-2 levels were lower in patients with advanced stages (T3+T4, AJCC III-IV), histologic grade > 3, and tumor size ≥5 cm. Kaplan-Meier analysis revealed a higher overall survival rate in the patients with a high CLEC-2 levels than in those with a low CLEC-2 levels (p = 0.035). Univariate Cox analysis showed that a high CLEC-2 level was independently associated with better overall survival. Spearman correlation analysis showed that CLEC-2 plasma levels were positively correlated with stages T0-T2, grades 1-2, ALT, APRI, and tumor size <2 cm, but negatively correlated with platelet count.

**Conclusion:** Our findings suggest that lower plasma CLEC-2 levels are associated with advanced breast cancer features. CLEC-2 is significantly associated with breast cancer prognosis and may serve as a prognostic marker in patients with breast cancer.

## Introduction

Recent epidemiological data indicate a steady rise in breast cancer incidence, with a 1% annual increase from 2012 to 2021 1. The most notable rise is among younger women, especially Asian American/Pacific Islanders, with a 2.7% and 2.5% yearly increase, respectively 1. Data from the US estimated that 56,500 cases of ductal carcinoma *in situ* and 310,720 new cases of invasive breast cancer would be diagnosed in women in 2024, along with 42,250 deaths due to breast cancer^1^. In Taiwan in 2018, the breast cancer prevalence among women aged 45-69 was 188-194 per 100,000, with 16,988 new cases (including ductal carcinoma *in situ* and invasive cancer) and 2,418 deaths. By 2020, the age-adjusted incidence rate was 47.8 per 100,000, with a mortality rate of 13.6 per 100,000 2. A study conducted from 2010 to 2020 highlighted improved survival rates for cancers identified through screening mammography 3. However, even in patients who undergo surgical treatment, some still develop postoperative metastasis, side effects of radiotherapy, and drug resistance. This is likely due to an incomplete understanding of breast cancer pathogenesis and the drug action mechanisms. Consequently, there is a crucial need to identify novel biomarkers and elucidate the underlying mechanisms to improve prognosis and optimize the effectiveness of breast cancer treatment.

C-type lectin-like receptor 2 (CLEC-2), first identified in 2006, is a type II transmembrane receptor that contains one or more C-type lectin-like domains and belongs to the C-type lectin superfamily 4. It functions as a platelet-associated molecule, and acts as an activation receptor for both the endogenous ligand podoplanin and snake venom toxin rhodocytin 5,6. CLEC-2 has been shown to play critical roles in immune responses, tumor cell-induced platelet aggregation, and blood-lymphatic/vascular separation 7-9. Furthermore, CLEC-2 has been shown to suppress gastric cancer metastasis by preventing activation of the GSK3B and AKT signaling pathways 10,11. Suzuki-Inoue et al. demonstrated that CLEC-2 could inhibit the aggregation of platelets and metastasis of colon cancer 12. Moreover, Hu et al. showed that low CLEC1B expression combined with high PD-L1 expression was associated with poorer clinical outcomes in patients with hepatocellular carcinoma (HCC) 13. In addition, Liang et al. demonstrated the potential of CLEC1B as a prognostic biomarker for HCC, and that CLEC1B expression was associated with the infiltration of immune cells 14. Nevertheless, the relationships between clinicopathological characteristics and CLEC-2 concentrations in patients with breast cancer have yet to be clarified. Therefore, the aim of this study was to assess plasma CLEC-2 levels in patients with breast cancer and in a cancer-free control group. We also examined the associations between CLEC-2 concentrations and clinicopathological characteristics, as well as the potential of CLEC-2 as a prognostic biomarker.

## Methods

### Study subjects

This study included 98 women who had a new diagnosis of breast cancer and received surgery at our hospital from September 2023 to December 2024. The inclusion criteria were: (1) a diagnosis of invasive or malignant breast cancer; (2) treatment-naïve patients (including radiotherapy, immunotherapy and chemotherapy) who were scheduled to undergo partial mastectomy or mastectomy; and (3) patients who signed informed consent before study enrollment. The exclusion criteria were: (1) having previously received a mastectomy or been treated for cancer (immunotherapy, radiotherapy, or chemotherapy); and (2) those who did not provide informed consent. We also recruited 98 age-matched controls, who were women attending an annual health examination at our hospital and had no prior history of cancer and normal mammography findings. The control group also gave written informed consent to participate in the study, which was approved by the Institutional Review Board of E-Da Hospital (no. EMRP-112-082). Information of the patients was obtained from hospital medical records. The mean age of the included patients was 53 years (range: 24-86 years). Cancer stage was determined based on the American Joint Committee on Cancer (AJCC) classification. Obesity was defined according to the criteria of the Department of Health, Taiwan, as a body mass index (BMI) of ≥27 kg/m².

### Laboratory data

Antecubital vein peripheral blood samples were collected before receiving any cancer treatment. Peripheral blood was collected into EDTA-coated vacutainer tubes and processed within 2 hours of collection. Plasma levels of aspartate transaminase and alanine transaminase (ALT) were measured using a parallel multichannel analyzer (Hitachi 7170A, Tokyo, Japan), as described previously 15. Carcinoembryonic antigen (CEA) concentrations were assessed via chemiluminescent microparticle immunoassay. Prothrombin time was measured using the clotting method. Peripheral leukocyte analysis was conducted on an automated cell counter (XE-2100 Hematology Alpha Transportation System; Sysmex, Kobe, Japan), including total and differential leukocyte count (neutrophils, monocytes, and lymphocytes). Factors related to red blood cells such as mean corpuscular hemoglobin concentration, hematocrit and hemoglobin were also measured, along with red cell distribution width-standard deviation, width-coefficient of variation and platelet count. Absolute leukocyte subtype counts were calculated by multiplying their respective percentages by the total leukocyte count. Enzyme-linked immunosorbent assay (ELISA) (Wuhan Fine Biotech Co., Ltd. (FineTest), Hubei, China; catalog number EH4717) was performed to evaluate the concentrations of plasma CLEC-2 following the protocols of the manufacturer. This sandwich ELISA (double-antibody format) utilizes a pre-coated anti-CLEC1B capture antibody and a biotinylated anti-CLEC1B detection antibody, as described by the manufacturer. Although the ELISA kit is labeled as detecting CLEC1B, it targets the soluble CLEC-2 protein encoded by the CLEC1B gene, in accordance with standard nomenclature. Dilution and standard curves were parallel, and the intra- and inter-assay coefficients of variation of the assay were 4.34 to 4.73% (n = 3) and 4.95 to 6.42% (n = 3), respectively. Each sample was measured twice in one experiment.

### Clinicopathologic characteristics of the tumors

The presence of breast cancer was confirmed by IHC staining for estrogen receptors (ERs) and progesterone receptors (PRs). Staging was based on the TNM system, and the Bloom-Richardson system was used to grade histology. All participants were classified into the following subgroups: age (< 50 or ≥ 50 years), Ki67 level (< 14% or ≥14%), AJCC stage (0-II or III-IV), histological grade (1+2 or > 3), pathologic T stage (T0+T1+T2 or T3+T4), lymph node metastasis (N0+N1 or N2+N3), size of the tumor (< 2, 2-5, or > 5 cm), ER status (-/+), PR status (-/+), and human epidermal growth factor receptor (HER2) status (-/+). IHC analysis of Ki-67, HER2, PR and ER were used to determine the molecular tumor subtype 16, which included triple-negative, HER2-enriched, luminal A, luminal B HER2-negative, and luminal B HER2-positive. Luminal A and B subtypes were defined as described previously 16. Luminal A was defined as ≥ 20% ER/PR positivity, low Ki-67 level (< 14%), and HER2 negativity; luminal B-like subtype with HER2 negativity was characterized by ER positivity, HER2 negativity, along with either a high Ki-67 expression (≥ 14%) or negative or low PR expression (< 20%); and luminal B-like subtype with HER2 positivity was characterized by ER positivity, the overexpression or amplification of HER2, and the presence of any level of Ki-67 and PR.

### Spike-in experiment for interference analysis with recombinant podoplanin

According to a previous report 17, serum podoplanin concentrations in cancer patients (n = 284) ranged from 0.52 to 185.40 ng/ml. Based on these findings, recombinant podoplanin at a final concentration of 200 ng/ml was included for interference analysis. For this purpose, the standard curve was prepared in two conditions: one supplemented with 10 μL PBS and the other with 10 μL of recombinant podoplanin (200 ng/ml). In addition, serum samples from 16 breast cancer patients and matched controls were individually treated with either 10 μL PBS or 10 μL of recombinant podoplanin (200 ng/ml). All experiments were conducted in duplicate.

### Statistical analysis

Descriptive statistics were used to summarize the results. The Kolmogorov-Smirnov test was applied to assess normality of the data, while Levene's test was applied to evaluate the homogeneity of variance. Continuous variables were presented as mean ± standard deviation, and differences between groups were assessed with either the unpaired Student's *t*-test or Wilcoxon rank-sum test. One-way analysis of variance (ANOVA), followed by Tukey's pairwise comparison, was used to evaluate differences in CLEC-2 levels among non-breast cancer controls, molecular tumor subtypes, and tumor size groups. Categorical variables were presented as frequencies (percentages). The relationships between plasma CLEC-2 levels and other variables were evaluated using Spearman's rank correlation analysis. Kaplan-Meier survival analysis was performed, and differences between groups were determined using the log-rank test. Univariate Cox proportional hazard analysis was used to calculate hazard ratio (HR) with 95% confidence interval (CI) to identify independent predictors of survival. A p-value of less than 0.05 was considered statistically significant. All statistical analyses were conducted using JMP version 10.0 for Windows (SAS Institute, Cary, NC, USA).

## Results

### Clinicopathological characteristics of the patients

Table 1 shows the clinicopathological characteristics of the patients, of whom 28.6% had hypertension, 4.1% had diabetes mellitus, and 16.3% had hyperlipidemia; none of the patients were immunosuppressed. Table 1 also shows that 18.4% of the patients were classified as having a pathologic T stage of T3 or T4, 15.3% presented with lymph node metastases classified as N2 or N3, and 66.3% had tumors measuring ≥ 2 cm. Regarding the molecular tumor subtype, luminal A accounted for 33.7% of cases, luminal B HER2-negative for 27.6%, luminal B HER2-positive for 15.3%, HER2-enriched for 6.1%, and triple-negative for 17.3%.

### Plasma CLEC-2 concentrations

**Figure 1:**
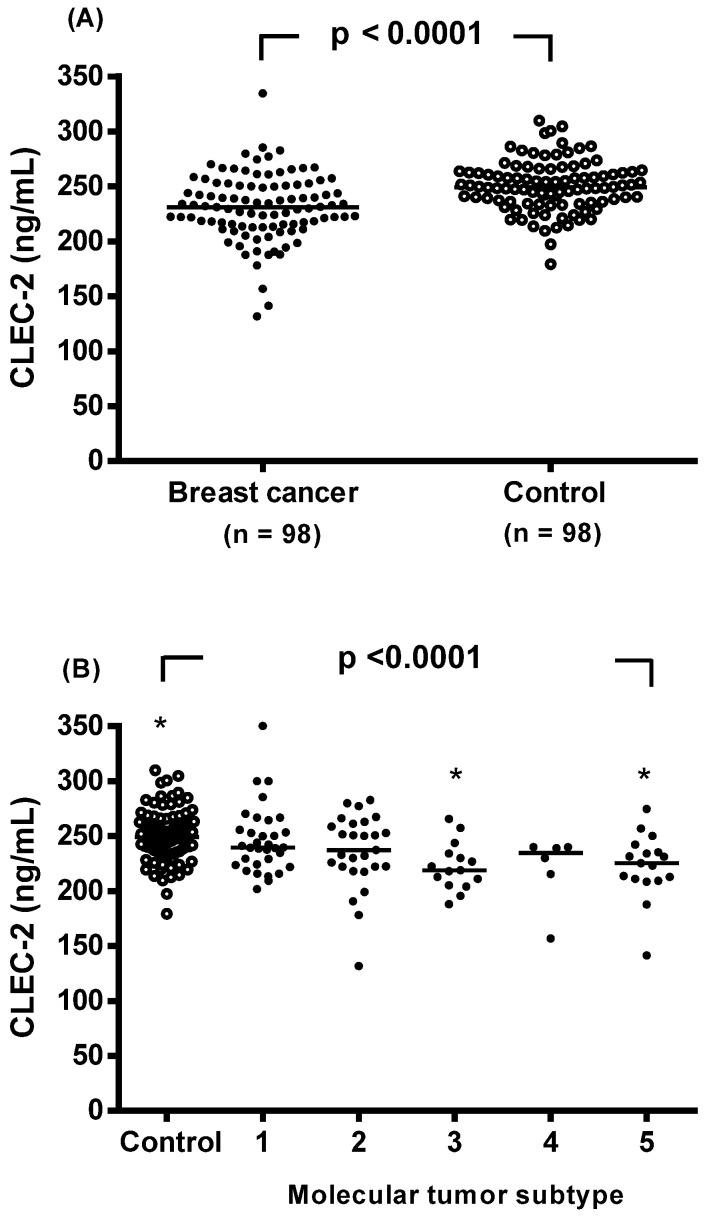
Individual plasma levels of C-type lectin-like receptor 2 (CLEC-2) were assessed based on disease status. (A) Subjects were categorized as either breast cancer patients or non-breast cancer controls. Plasma CLEC-2 levels were compared between the two groups using an unpaired Student's t-test. (B) Plasma CLEC-2 levels were further analyzed in non-breast cancer controls and in breast cancer patients stratified by molecular tumor subtype. The dot plot illustrates plasma CLEC-2 concentrations in non-breast cancer controls and in patients with five molecular tumor subtypes of breast cancer: (1) luminal A (n = 33), (2) luminal B HER2-negative (n = 27), (3) luminal B HER2-positive (n = 15), (4) HER2-enriched (n = 6), and (5) triple-negative (n = 17). Statistical differences among groups were assessed using one-way analysis of variance (ANOVA), followed by Tukey's pairwise comparison test. Data are presented as individual values and median concentrations (ng/mL). The horizontal line across the individual values indicates the median. p < 0.0001 vs. control group by ANOVA with Tukey's test. Asterisks indicate statistically significant differences compared to control.

The CLEC-2 concentration was significantly reduced in the breast cancer patients compared to the controls, with a median value (interquartile range) of 231.2 (213.5-250.9) ng/mL compared to 249.2 (235.8-263.4) ng/mL, respectively (p < 0.0001, Figure 1A). Plasma CLEC-2 levels were further analyzed in non-breast cancer controls and in breast cancer patients stratified by molecular tumor subtype (Figure 1B). CLEC-2 levels in the luminal B HER2-positive and triple-negative subtypes were significantly lower compared to non-breast cancer controls (222.8 ± 24.9 and 222.2 ± 33.4 vs. 250.3 ± 23.0, respectively; p <0.0001).

### Plasma CLEC-2 concentration and clinicopathologic markers

The CLEC-2 concentration was higher in the breast cancer patients with a tumor size < 2 cm, pathologic stage T0, T1, or T2, histologic grade 1 or 2, and AJCC stages 0-II. However, no significant associations were observed between CLEC-2 levels and age (< 50 vs. ≥ 50), lymph node metastasis (N2+N3 vs. N0+N1), Ki67 status (< 14% vs. ≥ 14%), HER2/ER/PR status (positive vs. negative), or molecular tumor subtype (luminal A, luminal B HER2-negative, luminal B HER2-positive, HER2-enriched, or triple-negative), as all p-values were > 0.05 (Table 2).

### Correlations between clinical and biochemical variables and CLEC-2 concentration

Spearman correlation analysis revealed that CLEC-2 plasma concentration showed significant positive correlations with tumor size < 2 cm, pathologic stages T0/T1/T2, histologic grades 1-2, ALT, and APRI, whereas a significant negative correlation was identified with platelet count (Table 3).

### Association of CLEC-2 concentration with overall survival

**Figure 2:**
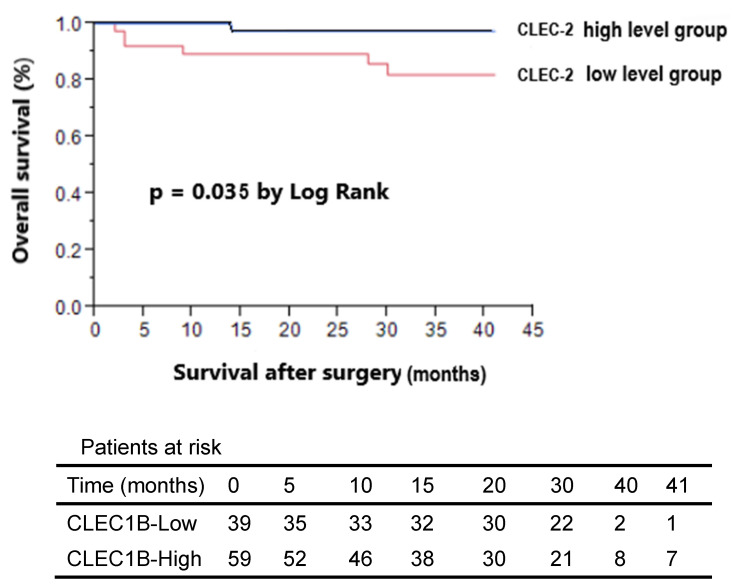
Kaplan-Meier survival analysis of breast cancer patients stratified by plasma C-type lectin-like receptor 2 (CLEC-2) levels. Patients were categorized into high (n = 59) and low (n = 39) CLEC-2 level groups based on the median plasma CLEC-2 concentration (223 ng/mL). Overall survival after surgery was significantly better in the high CLEC-2 group compared to the low CLEC-2 group (p = 0.035, log-rank test). The number of patients at risk at each time point is shown below the survival curves.

In the analysis of overall survival stratified by plasma CLEC-2 level, patients were dichotomized into high and low CLEC-2 level groups based on the median plasma CLEC-2 concentration (223 ng/mL). Patients with high CLEC-2 levels had a significantly better overall survival rate (Figure 2). Univariate analysis identified low CLEC-2 plasma level, tumor size > 5 cm, pathologic T stage (T3+T4), histologic grade > 3, and AJCC stage III-IV as significant prognostic factors associated with poor overall survival. However, in an exploratory subgroup analysis focusing on age, age ≥ 50 years was not significantly associated with poorer overall survival (Table 4) and should be interpreted separately from the main cohort analysis.

### Spike-in experiment with recombinant podoplanin

**Figure 3:**
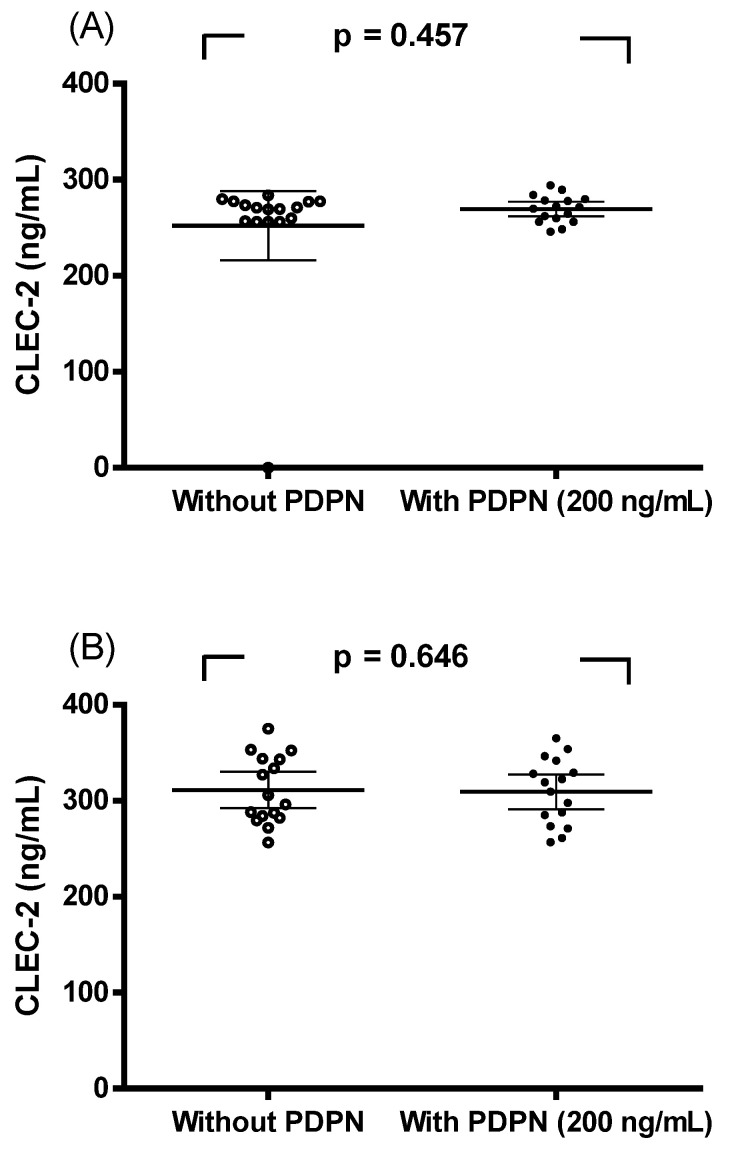
Spike-in experiment with recombinant podoplanin (PDPN). CLEC-2 concentrations in serum from breast cancer patients (A) and controls (B) with or without recombinant PDPN (200 ng/ml). No significant differences were detected by paired sample t-tests (A, p = 0.457; B, p = 0.646).

A spike-in assay using recombinant podoplanin (200 ng/ml) was conducted. CLEC-2 concentrations in paired serum samples were compared in the presence or absence of podoplanin. As shown in Figure 3, no significant differences were observed in breast cancer patients (Figure 3A, p = 0.457) or in controls (Figure 3B, p = 0.646) when analyzed by paired sample *t*-tests. These results indicate that recombinant podoplanin did not interfere with CLEC-2 measurements in either group.

## Discussion

This study investigated the clinical significance of plasma CLEC-2 concentrations in patients with breast cancer. There were three key findings regarding CLEC-2 and breast cancer in this study. (1) Plasma CLEC-2 levels were lower in the breast cancer patients compared to the controls, particularly in the patients with a larger tumor size (> 5 cm), advanced stage (AJCC III-IV), pathologic T stage (T3+T4), and high histologic grade. In addition, CLEC-2 levels in the luminal B HER2-positive and triple-negative subtypes were significantly lower than in non-breast cancer controls. (2) Higher plasma CLEC-2 levels were positively correlated with smaller tumor size (< 2 cm), early-stage (T0-T2), lower-grade tumors (grades 1-2), ALT and APRI, but negatively correlated with platelet count. (3) Higher plasma CLEC-2 levels were associated with better overall survival, as shown by Kaplan-Meier and univariate analyses. These findings suggest that CLEC-2 may be a useful non-invasive biomarker reflecting both disease severity and prognosis in breast cancer. While Etemad et al. 18 reported low plasma CLEC-2 levels across various tumor types, to our knowledge, our study is among the first to specifically demonstrate reduced plasma CLEC-2 levels in patients with breast cancer, with a particular focus on their association with clinicopathological features and survival outcomes.

The first key finding of the significant reduction in plasma CLEC-2 levels in the breast cancer patients compared to the controls (Figure 1) is consistent with findings in other malignancies, and supports its potential tumor-suppressive role 13,14,19. Furthermore, CLEC-2 levels were significantly lower in the luminal B HER2-positive and triple-negative subtypes compared to controls, aligning with the hypothesis that more aggressive breast cancer subtypes are associated with reduced CLEC-2 expression. Notably, lower CLEC-2 levels were observed in the patients with larger tumors (>5 cm), advanced stage (AJCC III-IV), pathologic T stage (T3+T4), and high histologic grade, suggesting its relevance as a marker of disease severity (Table 2). Accumulating evidence highlights the critical role of bidirectional interactions between platelets and tumor cells in promoting cancer progression and metastasis 20. The binding of CLEC-2 to podoplanin expressed on tumor cells triggers platelet activation, aggregation, and the release of bioactive molecules that support tumor cell survival, adhesion to the vascular endothelium, extravasation to distant metastatic sites, and subsequent tumor growth 21-23. In parallel, the clustering of podoplanin by platelet CLEC-2 modulates multiple signaling pathways that regulate tumor cell migration and invasion 12,21. However, CLEC-2 has been shown to exert tumor-suppressive effects in gastric cancer by inhibiting signaling pathways such as AKT and glycogen synthase kinase-3 beta, thereby reducing tumor invasiveness 10,11. In addition, Zhang et al. 24 reported that overexpression of CLEC-2 suppresses the proliferation and migration of Huh7 cells. Despite its known functions in other cancers, research on CLEC-2 in breast cancer remains limited. Understanding its dual role could provide insights into breast cancer progression and potential therapeutic targets.

The second major finding is that higher plasma CLEC-2 levels were positively correlated with smaller tumor size (<2 cm), early tumor stage (T0-T2), lower tumor grade (1-2), ALT and APRI, but negatively correlated with platelet count (Table 3). These findings underscore the potential of CLEC-2 as a non-invasive biomarker for early detection and disease monitoring in breast cancer. According to Liang et al. CLEC1B shows a significant association with immune cell subsets and may impact the effectiveness of immunotherapy in HCC patients 14. Furthermore, CLEC-2, a C-type lectin-like receptor, serves as the receptor for rhodocytin, a snake venom protein that activates platelets. It initiates strong platelet activation signals via Src and Syk kinases and PLCγ2, similar to the signaling cascade of the GPVI/FcRγ-chain collagen receptor complex 25. Moreover, previous studies have shown that CLEC1B may act as a tumor suppressor in liver cancer, being involved in the regulation of tumor proliferation and metastasis 24,26. The downregulation of CLEC1B has been correlated with unfavorable clinical outcomes in HCC 13,14,19. In our study, CLEC-2 levels were positively correlated with ALT and APRI. Although CLEC-2 is not a conventional liver function marker (like alanine transaminase and aspartate transaminase), this association may reflect systemic inflammation or liver-related immune activity in the context of cancer. Additionally, the inverse correlation with platelet count warrants further investigation, given CLEC-2's known role in platelet biology. Interestingly, although CLEC-2 is primarily expressed on platelets and is known to be released upon platelet activation, the observed reduction in its plasma concentration among patients with breast cancer appears counterintuitive. Several potential mechanisms may account for this paradox. First, CLEC-2 functions as a high-affinity receptor for podoplanin, a transmembrane glycoprotein that is frequently upregulated in various malignancies, including advanced-stage breast cancer 27. The interaction between CLEC-2 and podoplanin promotes platelet aggregation induced by tumor cells and has been implicated in tumor metastasis. Elevated podoplanin expression within the tumor microenvironment may result in the local sequestration of CLEC-2, either via stable ligand-receptor binding or receptor internalization at the tumor-platelet interface, ultimately decreasing the levels of free, circulating CLEC-2 detectable in plasma. Moreover, in patients with higher tumor burden, circulating or tissue-associated podoplanin may form complexes with CLEC-2 that sterically hinder antibody recognition in sandwich ELISA assays. This "epitope masking" effect may lead to an underestimation of soluble CLEC-2 levels in plasma, particularly in advanced disease stages 27. To directly examine this possibility, we performed a spike-in assay using recombinant podoplanin (200 ng/ml) and compared CLEC-2 concentrations in paired serum samples with or without podoplanin. As shown in Figure 3, no significant differences were observed in either breast cancer patients (Figure 3A, p = 0.457) or controls (Figure 3B, p = 0.646). These results indicate that recombinant podoplanin did not interfere with CLEC-2 quantification in our assay system, suggesting that epitope masking is unlikely to fully explain the observed reduction of plasma CLEC-2 levels in breast cancer patients. Second, chronic platelet activation in the tumor milieu may lead to CLEC-2 downregulation through receptor shedding or internalization, thereby diminishing the pool of CLEC-2 available for release into the bloodstream 5. Such mechanisms could contribute to reduced systemic levels, even in the presence of ongoing platelet activation. Thirdly, although CLEC-2 expression has been documented in murine neutrophils and macrophages 28, its presence in human leukocytes remains to be definitively established. CLEC-2 expressions other than leukocyte-source should be investigated more deeply to explain our observation. Collectively, these observations suggest that the reduced plasma soluble CLEC-2 levels observed in breast cancer may reflect a multifactorial process, potentially involving ligand-mediated sequestration, receptor downregulation, assay interference, and limited expression from alternative cellular sources. Importantly, decreased circulating soluble CLEC-2 should not be interpreted solely as an indicator of reduced production or release. To further elucidate the regulatory dynamics of CLEC-2 in the context of breast cancer progression, future mechanistic studies should incorporate tumor tissue immunohistochemistry along with simultaneous quantification of plasma podoplanin levels.

The fourth key finding of this study is that Kaplan-Meier analysis and univariate Cox regression revealed a significant association between higher plasma CLEC-2 levels and improved overall survival, suggesting that CLEC-2 may serve as a favorable prognostic biomarker in breast cancer (Table 4 and Figure 2). This result is consistent with previous studies that have identified CLEC-2 as a prognostic biomarker in HCC 13,14,19. We therefore propose that CLEC-2 may also play a critical role in breast cancer prognosis. Supporting this hypothesis, emerging evidence indicates that: (1) CLEC-2 exerts an inhibitory effect on platelet aggregation 29; (2) it is significantly downregulated in HCC 30; and (3) it is involved in the metastasis of various cancer types 21.

Although this study offers important insights, several limitations should also be discussed. First, as the sample size was relatively small, future studies with a larger cohort are needed to strengthen these findings. Second, the cross-sectional nature of the data limits causal inference, and dynamic changes in CLEC-2 levels during treatment or disease progression were not assessed. Third, CLEC-2 levels were assessed solely using ELISA, and the use of complementary methodologies may provide a more comprehensive understanding of its role in breast cancer. Fourth, in advanced stages of disease, the masking effect between podoplanin and CLEC-2 has been proposed as a potential source of underestimation of free soluble CLEC-2 levels in plasma, particularly in patients with higher tumor burden or increased podoplanin expression. In our study, however, a spike-in assay using recombinant podoplanin (200 ng/ml) demonstrated no significant interference with CLEC-2 quantification in either breast cancer patients or controls (Figure 3). These findings suggest that epitope masking is unlikely to fully explain the observed reduction of plasma CLEC-2 levels. Nevertheless, future investigations should include the assessment of circulating podoplanin levels and explore additional mechanisms that may affect CLEC-2 detectability and regulation in the cancer setting. Finally, we did not elucidate mechanistic insights into the function of CLEC-2 in breast cancer pathogenesis. We acknowledge that if CLEC-2 expression were indeed induced in breast tumor tissue, an increase in circulating soluble CLEC-2, particularly in early-stage disease, would be expected. Interestingly, our findings are consistent with this notion, as higher plasma CLEC-2 levels were observed in patients with smaller tumors, lower histologic grade, and AJCC stages 0-II (Table 2). In addition, although CLEC-2 has been reported to be expressed in certain other tumor types, data from the Human Protein Atlas show no detectable CLEC1B expression in either normal breast tissue or commonly studied human breast cancer cell lines. Thus, our original hypothesis regarding CLEC-2 involvement in breast cancer is not directly supported by available transcriptomic or proteomic data and should be interpreted with caution. Future studies should further investigate this aspect using large-scale cancer genome databases such as the Gene Expression Omnibus and/or The Cancer Genome Atlas, or through *in vitro* experiments employing well-characterized breast cancer cell lines, including MDA-MB-231, MCF-7, T-47D, and SK-BR-3.

## Conclusions

Our findings indicate that lower plasma levels of CLEC-2 are associated with more advanced disease stages and poorer prognosis in patients with breast cancer. These results provide preliminary evidence supporting CLEC-2 as a potential circulating biomarker of tumor burden and survival. Further prospective and mechanistic studies are warranted to validate its clinical utility and to elucidate its functional role in breast cancer progression.

## Figures and Tables

**Table 1:** The clinicopathological characteristics of the 98 patients diagnosed with breast cancer.

Parameter	Number	Percentage (%)
Age (years)		
< 50	45	45.9
≥ 50	53	54.1
Range	24-86	
Mean± SD	53.2±13.2	
Obesity	32	32.7
Menstrual status		
Pre-menopause	47	48.0
Post-menopause	51	52.0
Comorbidities		
Hypertension	28	28.6
Diabetes mellitus	4	4.1
Hyperlipidemia	16	16.3
Tumor size (cm)		
< 2	33	33.7
2-5	48	49.0
> 5	17	17.3
Pathologic T stage		
T0+T1+T2	80	81.6
T3+T4	18	18.4
Lymph node metastasis		
N0+N1	83	84.7
N2+N3	15	15.3
Histologic grade		
1+2	52	53.1
> 3	46	46.9
AJCC Stage		
0-II	73	74.5
III-IV	25	25.5
Ki67 status		
< 14%	39	39.8
≥ 14%	59	60.2
Estrogen receptor		
Negative	18	18.4
Positive	80	81.6
Progesterone receptor		
Negative	33	33.7
Positive	65	66.3
HER2		
Negative	75	76.5
Positive	23	23.5
Molecular tumor subtype		
1 (Luminal A)	33	33.7
2 (Luminal B HER2-negative)	27	27.6
3 (Luminal B HER2-positive)	15	15.3
4 (HER2-enriched)	6	6.1
5 (Triple-negative)	17	17.3

AJCC, American Joint Committee on Cancer.

**Table 2:** Plasma concentration of C-type lectin-like receptor 2 grouped according to categorical variables.

Parameter	Number	CLEC-2 (ng/mL) (mean ± SD)	p-value
Age (years)			
< 50	45	226.8±23.5	0.208
≥ 50	53	239.6±63.5	
Tumor size (cm)			
< 2	33	234.4±25.3	0.002
2-5	48	233.5±25.3	
> 5	17	206.9±36.4	
Pathologic T stage			
T0+T1+T2	80	233.6±26.7	0.001
T3+T4	18	208.5±27.9	
Lymph node metastasis			
N0+N1	83	235.3±52.9	0.390
N2+N3	15	222.0±19.0	
Histologic grade			
1+2	52	243.3±61.8	0.027
> 3	46	223.4±34.5	
American Joint Committee on Cancer stage			
0-II	73	234.9±23.0	0.040
III-IV	25	221.5±32.8	
Ki67 status			
< 14%	39	244.2±70.8	0.113
≥ 14%	59	227.3±31.7	
Estrogen receptor			
Negative	18	221.5±33.3	0.148
Positive	80	236.8±52.8	
Progesterone receptor			
Negative	33	226.3±35.4	0.172
Positive	65	238.2±56.4	
HER2			
Negative	75	237.0±56.2	0.191
Positive	23	222.0±27.8	
Molecular tumor subtype			
Luminal A	33	246.6±77.8	0.398
Luminal B HER2-negative	27	235.3±34.1	
Luminal B HER2-positive	15	222.8±24.9	
HER2-enriched	6	220.3±35.7	
Triple-negative	17	222.2±33.4	

**Table 3:** Spearman correlation analysis of clinical and biochemical variables with expression and concentration of C-type lectin-like receptor 2.

Parameter	CLEC-2 plasma level	
	r	p-value
Tumor size (< 2 cm versus ≥ 2 cm)	0.342	0.001
Pathologic T stage (T0+T1+T2 versus T3+T4)	0.334	0.001
AJCC stage (0-II versus III-IV)	0.158	0.144
Ki67 (< 14% versus ≥ 14%)	0.111	0.297
Histologic grade (1+2 versus > 3)	0.216	0.040
Molecular tumor subtype (Luminal A versus luminal B HER2-negative, luminal B HER2-positive, HER2-enriched, and triple-negative)	0.096	0.373
AST	0.145	0.164
ALT	0.230	0.039
APRI	0.235	0.036
CEA	-0.147	0.152
Prothrombin time	-0.028	0.801
White blood cell count	-0.110	0.289
Neutrophil count	-0.221	0.099
Monocyte count	-0.252	0.059
Lymphocyte count	-0.061	0.650
Red blood cells	-0.134	0.197
Hemoglobin	0.018	0.862
Hematocrit	-0.007	0.945
MCH	0.181	0.080
MCHC	0.107	0.305
Platelet count	-0.235	0.022
RDW-SD	-0.008	0.937
RDW-CV	-0.134	0.195

CLEC-2, C-type lectin-like receptor 2; AJCC, American Joint Committee on Cancer; AST, Aspartate transaminase; ALT, Alanine transaminase; APRI, AST to platelet ratio index; CEA, carcinoembryonic Antigen; MCH, mean corpuscular hemoglobin; MCHC, mean corpuscular-hemoglobin concentration; RDW, red cell distribution width; SD, standard deviation; CV, coefficient of variation.

**Table 4:** Univariate analysis of factors affecting overall survival in the patients with breast cancer.

Variable	Hazard ratio	95% CI	p-value
Age: ≥ 50 years versus < 50 years	2.370	0.511-6.547	0.279
Tumor size: > 5 cm versus ≤ 5 cm	7.552	1.660-8.438	0.010
Pathologic T stage: T3+T4 versus T0+T1+T2	13.654	2.929-9.610	0.001
Histologic grade: > 3 versus 1+2	7.147	1.219-4.997	0.027
AJCC stage: III-IV versus 0-II	17.146	3.212-7.669	0.001
Plasma CLEC-2 levels: High versus low	0.141	0.007-0.833	0.029

AJCC, American Joint Committee on Cancer; CLEC-2, C-type lectin-like receptor 2.
